# MicroRNA let-7f-5p Inhibits Porcine Reproductive and Respiratory Syndrome Virus by Targeting MYH9

**DOI:** 10.1038/srep34332

**Published:** 2016-09-30

**Authors:** Na Li, Taofeng Du, Yunhuan Yan, Angke Zhang, Jiming Gao, Gaopeng Hou, Shuqi Xiao, En-Min Zhou

**Affiliations:** 1 College of Veterinary Medicine, Northwest A&F University, No. 22 Xinong Road, Yangling, Shaanxi, 712100, China; 2 Experimental Station of Veterinary Pharmacology and Veterinary Biotechnology, Ministry of Agriculture, China, No. 22 Xinong Road, Yangling, Shaanxi, 712100, China; 3 Institute of Immunology, Taishan Medical University, Taian, Shandong, 271000, China

## Abstract

Porcine reproductive and respiratory syndrome virus (PRRSV) is one of the most important viral pathogens in the swine industry. Current antiviral strategies do not effectively prevent and control PRRSV. Recent reports show that microRNAs (miRNAs) play vital roles in viral infections by post transcriptionally regulating the expression of viral or host genes. Our previous research showed that non-muscle myosin heavy chain 9 (MYH9) is an essential factor for PRRSV infection. Using bioinformatic prediction and experimental verification, we demonstrate that MYH9 expression is regulated by the miRNA let-7f-5p, which binds to the MYH9 mRNA 3′UTR and may play an important role during PRRSV infection. To understand how let-7f-5p regulates PRRSV infection, we analyzed the expression pattern of both let-7f-5p and MYH9 in porcine alveolar macrophages (PAMs) after infection with either highly pathogenic PRRSV (HP-PRRSV) or classical type PRRSV (N-PRRSV) using a deep sequencing approach with quantitative real-time PCR validation. Our results showed that both HP-PRRSV and N-PRRSV infection reduced let-7f-5p expression while also inducing MYH9 expression. Furthermore, let-7f-5p significantly inhibited PRRSV replication through suppression of MYH9 expression. These findings not only provide new insights into the pathogenesis of PRRSV, but also suggest potential new antiviral strategies against PRRSV infection.

Porcine reproductive and respiratory syndrome (PRRS) is one of the most highly infectious swine diseases^1^, resulting in great economic losses every year affecting the swine industry worldwide^2^. PRRS is characterized by respiratory disease, weight loss and poor growth performance, as well as late-term abortions. Porcine reproductive and respiratory syndrome virus (PRRSV), the causative agent of PRRS, is a single-stranded, positive-sense RNA virus of the genus *Arterivirus*, in the family *Arteriviridae* within the order *Nidovirales*^3^, and was identified both in Europe and the United States by the early 1990s^4^,^5^.

A highly pathogenic strain of PRRSV (HP-PRRSV), with a discontinuous deletion of 30 amino acids within its nonstructural protein 2 (NSP2), was associated with devastating atypical PRRS outbreaks in China in 2006. Characterized by high fever, high morbidity, and high mortality in pigs of all ages^6^, HP-PRRSV coexists in China with the long-established low pathogenic North American type PRRSV strains (N-PRRSV)^7^. Because existing antiviral strategies have been ineffective in providing sustainable protection, especially against highly pathogenic strains^8^,^9^, it is imperative that new measures are developed to control widespread virus infection.

PRRSV strains have a restricted repertoire of susceptible hosts, limited by a viral requirement for specific receptors on host cells^10^,^11^. Despite the restricted cell tropism of PRRSV, the virus is able to replicate in several non-permissive cell lines, indicating that additional proteins may facilitate virus entry^12^,^13^. Recently, non-muscle myosin heavy chain 9 (MYH9) was implicated in PRRSV infection. Through formation of intercellular nanotube connections that can transport infectious viral RNA from cell to cell, MYH9 facilitates intercellular viral spread, while allowing PRRSV to escape the host’s neutralizing antibody response^14^. Our previous work demonstrated that MYH9 also guides the infection process after virus particles attach to cell surface receptors, culminating in completion of subsequent un-coating events required for PRRSV genomic release within the host cell^15^.

MicroRNAs (miRNA) are a group of endogenous small noncoding RNAs which mediate posttranscriptional gene regulation by triggering mRNA degradation or blocking its translation^16^,^17^,^18^. Recruitment of miRNAs into the RNA-induced silencing complex (RISC) through base pairing between a 6 to 8 bp miRNA “seed region” with complementary sites on the 3′-untranslated region (3′-UTR) of target messenger RNAs (mRNAs) initiates specific downregulation of mRNA expression. Moreover, microRNA has been shown to participate not only in fundamental cell regulatory functions, but also in complex host-virus interactions^19^.

Recently, microRNAs have been reported to modulate PRRSV infection and replication in multiple ways, by targeting cellular genes essential for virus replication or directly targeting viral genomic sequences. Our previous work demonstrated that miR-24-3p suppressed cellular HO-1 expression, it concurrently promoted PRRSV replication^20^. Conversely, both miR-181 targeting of viral genomic RNA and cellular receptor CD163 have been implicated in the suppression of PRRSV replication^21^. Moreover, while miR-26 inhibits PRRSV replication by upregulating type I interferons^22^, miR-125b inhibits PRRSV replication by negatively regulating the NF-κB pathway^23^.

Here we investigated whether microRNAs exist that target MYH9 to alter regulation of PRRSV replication in PRRSV-permissive cells. Using bioinformatics prediction and experimental verification, we demonstrate that overexpression of let-7f-5p inhibits PRRSV replication in PAMs by targeting MYH9 sequences. PRRSV infection depressed let-7f-5p expression to promote MYH9 expression and facilitate viral replication. These findings not only provide an example of a host microRNA that modulates PRRSV replication, but also highlight the potential value of microRNA-mediated antiviral therapeutic strategies.

## Results

### Prediction and identification of the miRNAs which regulate the *MYH9* gene

Our previous studies indicate that MYH9 plays an important role in intercellular viral spread. In this study, we further investigated miRNAs which regulate MYH9 expression at the posttranscriptional level. MicroRNA prediction algorithms generally pair the “seed region” of the microRNA to a specific mRNA 3′ UTR sequence. To predict miRNAs which can bind to pig and monkey MYH9 3′UTRs, computer-based sequence analysis using RNAhybrid and Segal Lab online microRNA prediction tools were used. We found that one pig miRNA and three monkey miRNAs exhibited potential 3′UTR binding sites to pig and monkey MYH9 mRNAs, respectively. The miRNA identified as potentially targeting pig MYH9 mRNA was let-7f-5p, whereas miRNAs potentially targeting monkey MYH9 mRNA were let-7f-5p, mml-miR-638, and mml-miR-346.

**Figure 1:**
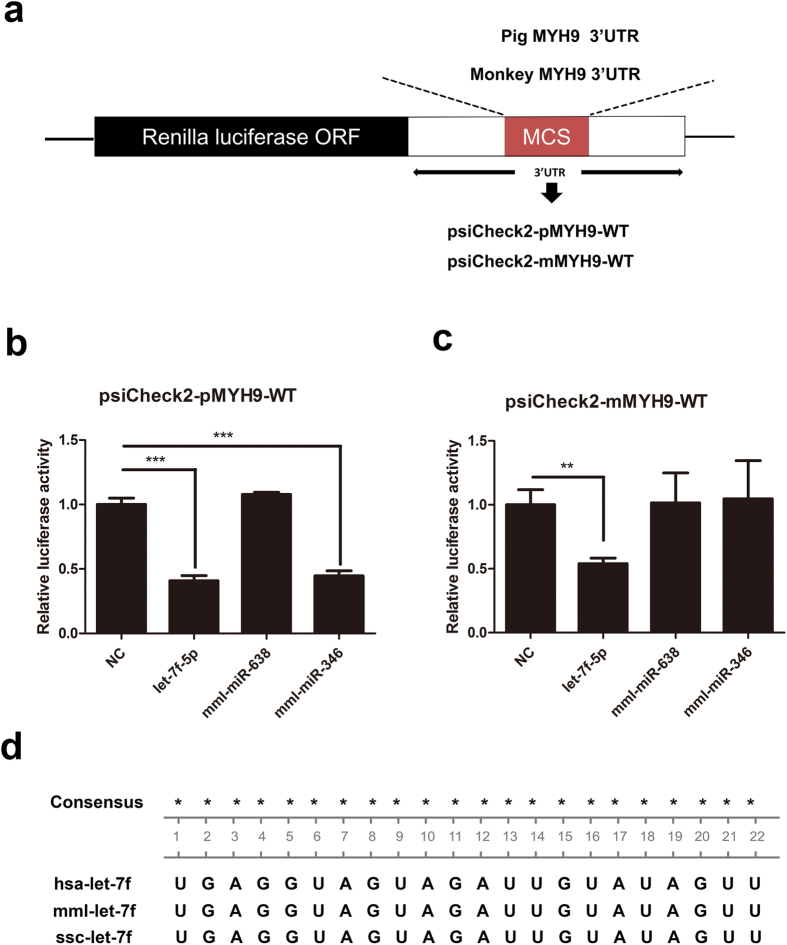
Luciferase reporter activity assay identifies let-7f-5p as a regulator of MYH9. (**a**) Pig MYH9 3′UTR and monkey MYH9 3′UTR were cloned into 3′UTR-luciferase reporter psiCheck2 vectors to create plasmids designated psiCheck2-pMYH9-WT and psiCheck2-mMYH9-WT, respectively. (**b**) HEK293FT cells were cotransfected with indicated mimics and psiCheck2-pMYH9-WT. (**c**) HEK293FT cells were cotransfected with indicated mimics and psiCheck2-mMYH9-WT. Thirty-six hrs later, cells were lysed and luciferase activity was measured. Results are expressed as mean ± SD of three independent experiments. *p* values were calculated using the Student’s *t* test. **P < 0.01, ***P < 0.001. (**d**) Sequence homology of let-7f-5p to human, monkey, and pig.

To address the question of whether the candidate miRNAs could potentially regulate MYH9 expression, the 3′ UTRs of pig and monkey MYH9 were cloned downstream of the luciferase ORF in a reporter vector (Fig. 1a). The reporter plasmids containing either pig MYH9 3′UTR (psiCheck2-pMYH9-WT) or monkey 3′UTR (psiCheck2-mMYH9-WT) were transfected into HEK293FT cells along with synthetic various miRNA mimics or a negative control mimics, and luciferase activity was measured. As shown in Fig. 1b, luciferase activity was downregulated in the presence of either let-7f-5p or mml-miR346 mimics, resulting in respective decreases of 62% and 58% in luciferase expression relative to psiCheck2-pMYH9-WT expression. Downregulation of luciferase activity was not observed with overexpression of mml-miR-638 or the negative control (NC). Only let-7f-5p mimics significantly decreased luciferase expression (by 56%) relative to psiCheck2-mMYH9-WT (Fig. 1c). Notably, let-7f-5p dramatically inhibited luciferase expression for both psiCheck2-pMYH9-WT (Fig. 1b) and psiCheck2-mMYH9-WT (Fig. 1c). These observations, coupled with the fact that let-7f-5p is well conserved among different host species (Fig. 1d), led us to choose let-7f-5p for further validation studies.

### Let-7f-5p, targeted to bind to the 3′UTR of MYH9, bound directly to the 3′UTR

**Figure 2:**
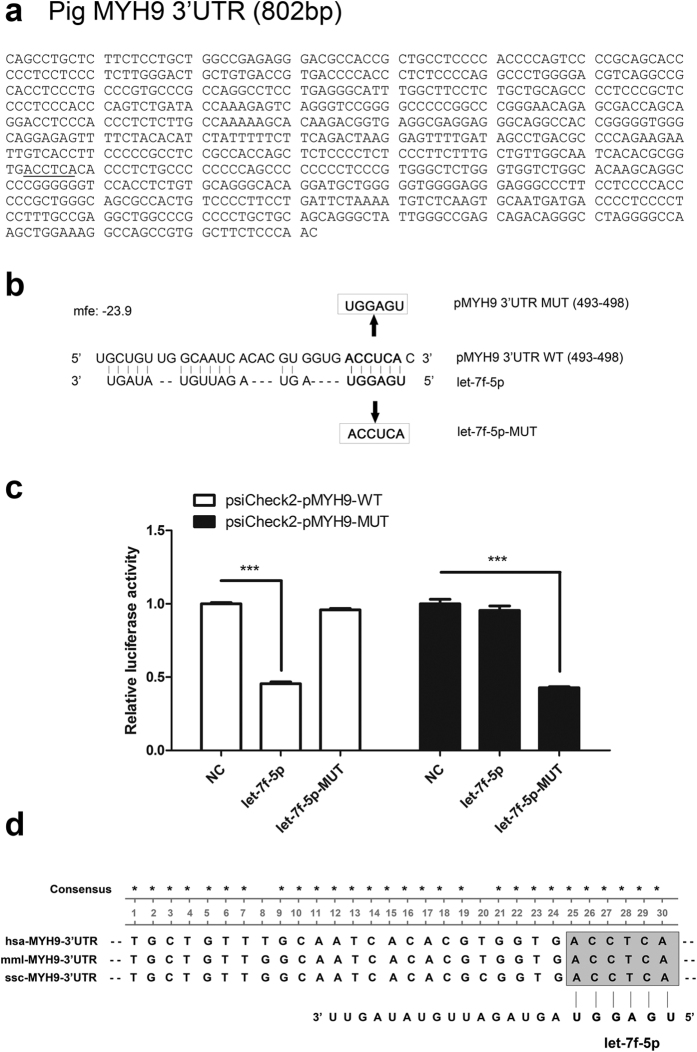
Let-7f-5p specifically binds to the 3′ UTR of pig MYH9. (**a**) The 3′UTR of pig MYH9 contains one seed-matched target site (highlighted) for let-7f-5p. (**b**) Schematic of the “seed region” match between let-7f-5p and pig MYH9 3′UTR. The mutation of six nucleotides in the seed match is shown. The positions of six seed match sites for let-7f-5p in the pig MYH9 3′UTR were replaced. (**c**) WT or MUT reporter plasmids were cotransfected with the let-7f-5p mimics, let-7f-5p-MUT mimics, or NC mimics into the HEK293FT cells. Reporter activities were determined thirty-six hrs post-transfection by dual-luciferase assays. Results are expressed as mean ± SD of three independent experiments. *p* values were calculated using Student’s *t* test. ***P < 0.001. (**d**) Sequence homology of the let-7f-5p binding sequence in the MYH9 3′UTR of human, monkey, and pig.

**Figure 3:**
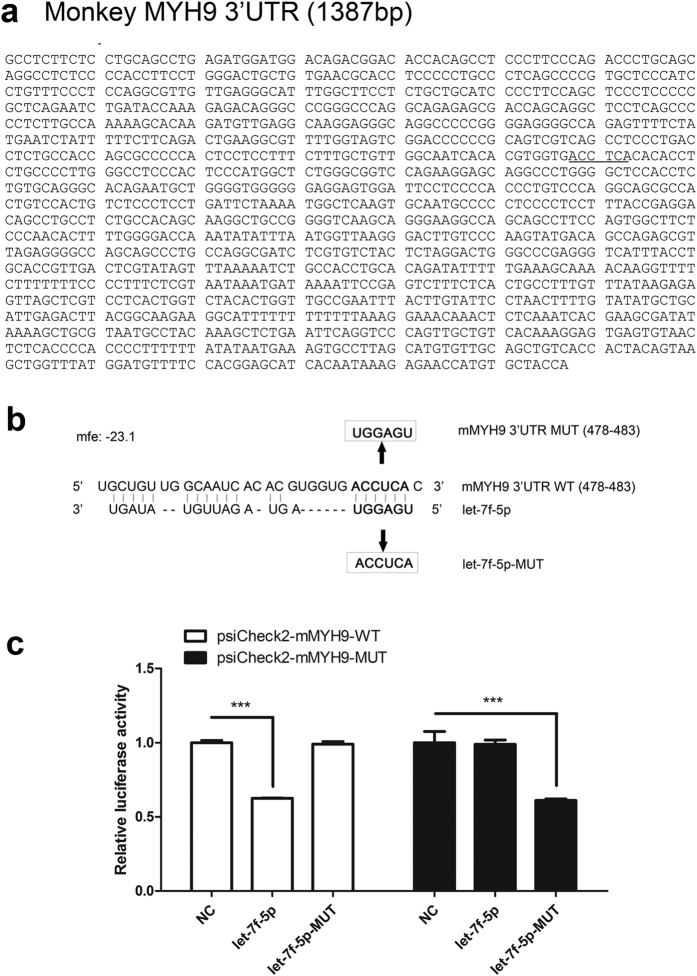
Let-7f-5p specifically binds to the 3′ UTR of monkey MYH9. (**a**) The 3′UTR of monkey MYH9 contains one seed matched target site (highlighted) for let-7f-5p. (**b**) Schematic of the “seed region” match between let-7f-5p and monkey MYH9 3′UTR. The mutation of six nucleotides in the seed match is shown. The positions of six seed match sites for let-7f-5p in the monkey MYH9 3′UTR were replaced. (**c**) WT or MUT reporter plasmids were cotransfected with the let-7f-5p mimics, let-7f-5p-MUT mimics, or NC mimics into HEK293FT cells. Reporter activities were determined thirty-six hrs post-transfection by dual-luciferase assays. Results are expressed as mean ± SD of three independent experiments. *p* values were calculated using Student’s *t* test. ***P < 0.001.

There are two putative MYH9 seed match sequences with perfect complementarity to let-7f-5p, each with an MFE of <–20 kcal/mol. Nucleotide regions spanning 493 to 498 nt of the pig MYH9 3′UTR (Fig. 2a) and 478 to 483 nt of the monkey MYH9 3′UTR (Fig. 3a) were predicted using RNAhybrid and are conserved across various species (Fig. 2d). To investigate whether let-7f-5p directly targets the MYH9 expression via binding to its mRNA 3′UTR, wild type (WT) target sequences and site-directed mutant sequences were cloned into the luciferase reporter vector (Figs 2b and 3b). Each of these plasmids was cotransfected with the indicated microRNA mimics into HEK293FT cells and subjected to the luciferase assay. As shown in Figs 2c and 3c, let-7f-5p significantly inhibited the luciferase activity of psiCheck2-pMYH9-WT (Fig. 2c left) and psiCheck2-mMYH9-WT (Fig. 3c left). However, disruption of the seed sequence in plasmids containing the mutated pig MYH9 3′UTR (psiCheck2-pMYH9-MUT) or the mutated monkey MYH9 3′UTR (psiCheck2-mMYH9-MUT) ablated the ability of let-7f-5p to inhibit the expression of luciferase (Figs 2c right and 3c right, respectively), supporting the view that let-7f-5p targeted MYH9 mRNA via specific binding to its 3′UTR. Similarly, the mutant let-7f-5p (let-7f-5p-MUT), which restored base complementarity with mutated MYH9 3′UTR sequences, did not block the luciferase expression from psiCheck2-pMYH9-WT and psiCheck2-mMYH9-WT (Figs 2c left and 3c left, respectively). In contrast, let-7f-5p-MUT significantly decreased the luciferase activity of psiCheck2-pMYH9-MUT and psiCheck2-mMYH9-MUT (Figs 2c right and 3c right, respectively). These results demonstrate that let-7f-5p directly targets the 3′UTR of MYH9 mRNA in a sequence-specific manner.

**Figure 4:**
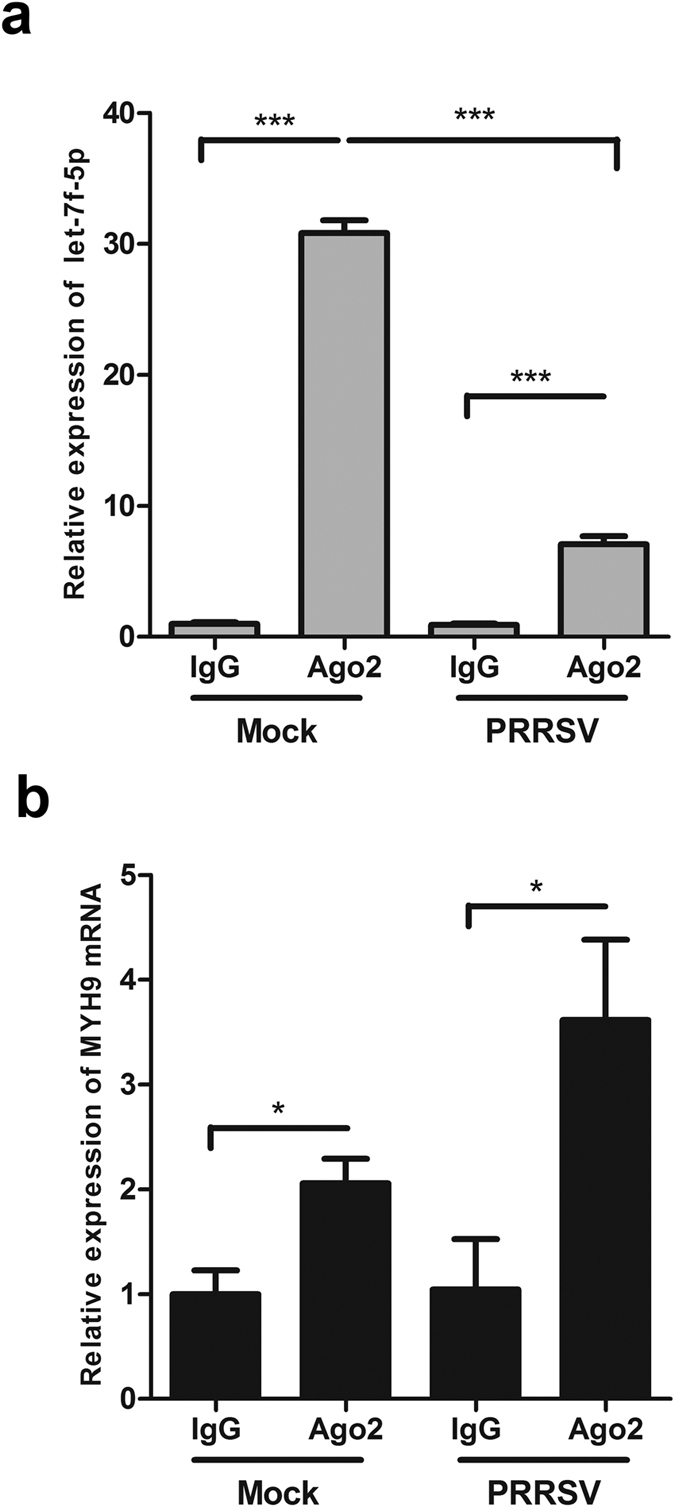
Let-7f-5p physically binds to MYH9 mRNA in the RISC. RNA of mock- and GD-HD-infected PAMs were subjected to Ago2-IP, the relative expression of let-7f-5p (**a**) and MYH9 mRNA (**b**) in the immunoprecipitates were determined by qRT-PCR. As a negative control, immunoprecipitation was performed using anti-Flag antibody beads (IgG). Results are expressed as mean ± SD of three independent experiments. *p* values were calculated using Student’s *t* test. *P < 0.05, ***P < 0.001.

To further demonstrate the direct interaction between let-7f-5p and MYH9 mRNA, immunoprecipitation (IP) analysis was carried out using anti-Ago2 antibody and cell lysates. PAMs were mock-infected or infected with the GD-HD strain at an MOI of 0.1. After 12 hours, Ago-2-bound entities within lysates from PRRSV-infected or non-infected PAMs were immunoprecipitated with anti-Ago2 monoclonal antibody. Next, let-7f-5p miRNA and MYH9 mRNA in the immunoprecipitates were detected by qRT-PCR. As shown in Fig. 4a, the let-7f-5p level associated with Ago2 protein was increased by 30.8-fold and 7.8-fold compared to that of the IgG isotype control in mock-infected and PRRSV-infected cells, respectively. However, the abundance of MYH9 mRNA increased by approximately 2.1-fold and 3.5-fold in mock-infected and PRRSV-infected cells, respectively, compared to that of the IgG isotype control (Fig. 4b). These results demonstrate that let-7f-5p directly interacts with MYH9 mRNA in the RISC.

### Let-7f-5p regulates MYH9 expression through both mRNA degradation and translational repression

**Figure 5:**
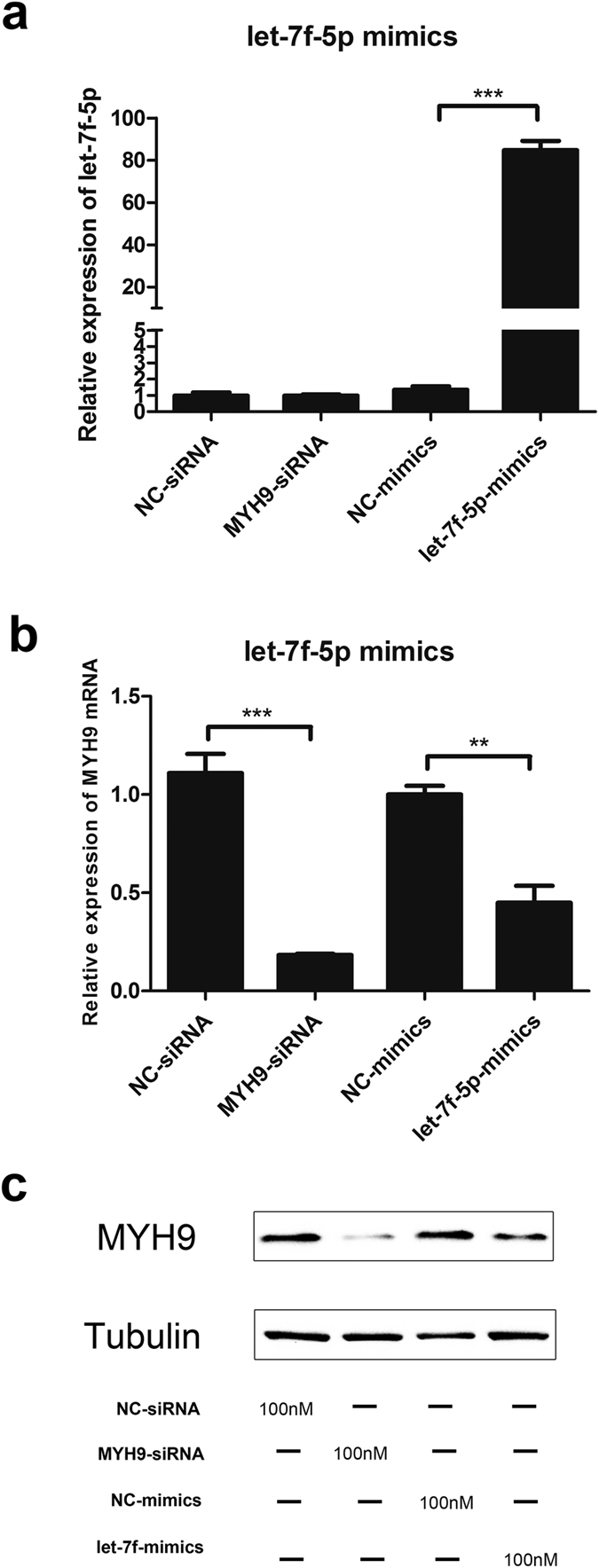
Overexpression of let-7f-5p decreases MYH9 mRNA and protein levels in PAMs. PAMs were transfected with 100nM of MYH9-siRNA, NC-siRNA, let-7f-5p mimics, and NC-mimics. Relative expression level of let-7f-5p (**a**) and MYH9 mRNA (**b**) in PAMs determined by qRT-PCR. MYH9 protein expression (**c**) analyzed by western blot with α-tubulin as the control. Results are expressed as mean ± SD of three independent experiments. *p* values were calculated using Student’s *t* test. **P < 0.01, ***P < 0.001.

To further investigate whether let-7f-5p can regulate MYH9 expression, PAMs were transfected with let-7f-5p, NC mimics, siMYH9 (as a positive control), or NC siRNA; the expression of let-7f-5p and MYH9 mRNA were examined at the indicated times. As shown in Fig. 5, expression of let-7f-5p in the various groups was examined by qRT-PCR and normalized against U6 rRNA expression (Fig. 5a). The expression level of MYH9 mRNA in cells transfected with the siMYH9 and let-7f-5p mimics were lower than that of the NC group. Notably, when PAMs were transfected with 100 nM let-7f-5p mimics for 24 h, the expression level of MYH9 mRNA decreased by 55% compared with that of negative controls (Fig. 5b). At the same time, expression of MYH9 protein in PAMs, which were transfected with let-7f-5p mimics, siMYH9, or negative controls for 36 h, was also detected by western blot. Moreover, MYH9 expression in cells treated with let-7f-5p mimics or siMYH9 was markedly reduced compared with negative control (Fig. 5c). Collectively, qRT-PCR and western blot assays revealed that let-7f-5p markedly inhibits the expression of MYH9 at both mRNA and protein levels.

**Figure 6:**
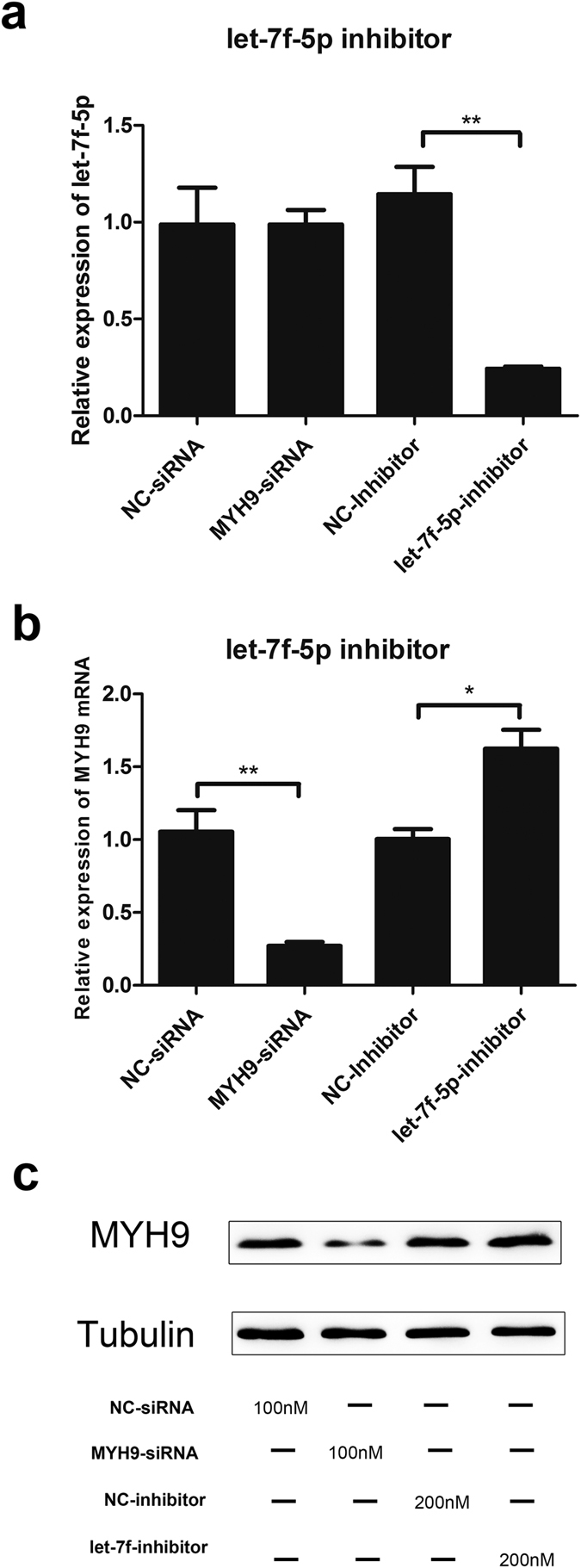
Inhibition of let-7f-5p enhances MYH9 mRNA and protein expression in PAMs. PAMs were transfected with indicated concentrations of MYH9-siRNA, NC-siRNA, let-7f-5p-inhibitor, and NC-inhibitor. Relative expression level of let-7f-5p (**a**) and MYH9 mRNA (**b**) in PAMs determined by qRT-PCR. MYH9 protein expression (**c**) analyzed by western blot, with α-tubulin as control. Results are expressed as mean ± SD of three independent experiments. *p* values were calculated using Student’s *t* test. *P < 0.05, **P < 0.01.

To confirm the effect of let-7f-5p on MYH9 expression, PAMs were transfected with let-7f-5p inhibitor and the negative control, then the expression of let-7f-5p and MYH9 were examined at the indicated times. As shown in Fig. 6, let-7f-5p inhibitor transfection led to a significant reduction in let-7f-5p mRNA expression level (Fig. 6a). When PAMs were transfected with 200 nM let-7f-5p inhibitor for 24 h, the expression level of MYH9 mRNA was increased by 62% compared with that of negative control (Fig. 6b). Similarly, western blotting showed that the level of MYH9 expression was increased by transfection of 200 nM let-7f-5p inhibitor relative to that achieved by transfection of a negative control inhibitor (Fig. 6c).

### A let-7f-5p decrease accompanied enhanced MYH9 expression in PRRSV-infected PAMs

**Figure 7:**
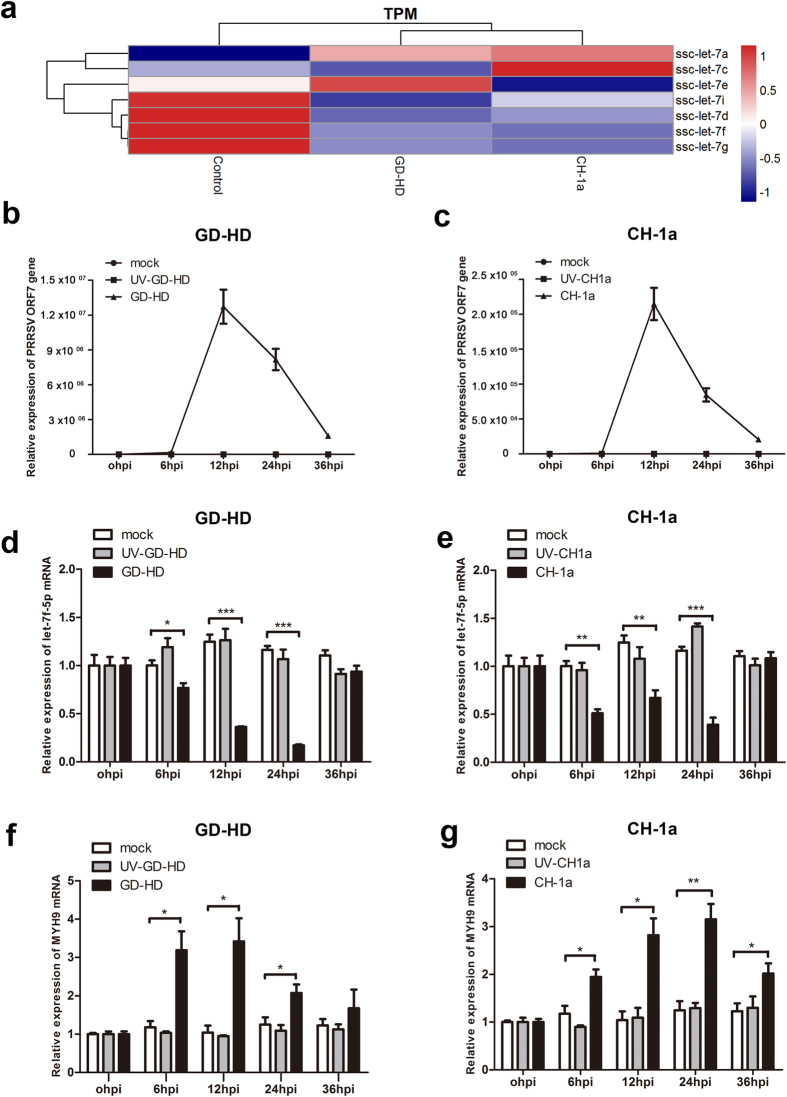
Let-7f-5p is reduced after PRRSV infection. (**a**) Heatmap for let-7 family members at 12 hpi compared with control group in the PAMs infected with two PRRSV strains, GD-HD and CH-1a (MOI = 0.1), quantified by microRNA deep sequencing. The log2 fold changes were used to plot the heatmap. PAMs cells were mock-infected or were infected with UV-inactivated or active forms of two PRRSV strains, GD-HD and CH-1a (MOI = 0.1). PRRSV ORF7 (**b,c**), let-7f-5p (**d,e**), and MYH9 (**f,g**) mRNA relative expression levels were determined using qRT-PCR. Results are expressed as mean ± SD of three independent experiments. p values were calculated using Student’s t test. *P < 0.05, **P < 0.01, ***P < 0.001.

In order to elucidate whether PRRSV infection affects the expression of let-7f-5p, microRNA let-7 family member expression in response to PRRSV infection was checked in PAMs. Small RNA deep sequencing was performed using libraries of RNA of mock-infected, HP-PRRSV (GD-HD)-, or N-PRRSV (CH-1a)-infected PAMs each at an MOI of 0.1 at 12 h post-infection (hpi). After deep sequencing data analysis, the numbers of let-7 family member reads of the three small RNA libraries were compared (Table 1). A striking observation was the downregulation of let-7f-5p miRNAs in both HP- and N-PRRSV-infected PAMs. Similar expression patterns were observed for several other let-7 miRNA members (Fig. 7a).

To further confirm the let-7f-5p expression profile associated with PRRSV infection, the levels of let-7f-5p in PAMs were detected using qRT-PCR. Total RNA was isolated from PAMs at the indicated time points and the PCR product of the ORF7 gene, a measure of the replication of the two PRRSV strains, was detected (Fig. 7b,c). Next, miRNA profiles were determined using a stem-loop-primer qRT-PCR. As shown in Fig. 7d,e, let-7f-5p was greatly reduced in PAMs infected with both PRRSV strains as early as 6 hpi and its expression remained lower in the PRRSV-infected PAMs than in mock and UV-inactivated virus controls at 12 hpi and 24 hpi.

We next asked the question of whether the downregulation of let-7f-5p was linked to the concomitant upregulation of expression of its target, MYH9 mRNA, in PRRSV-infected PAMs. To investigate this, mRNA profiles were generated from the same set of samples as those used for qRT-PCR. MYH9 was transcriptionally upregulated as early as 6 hpi; it reached peak expression at 12 hpi (3.29-fold), and then its expression decreased gradually as the HP-PRRSV infection progressed (Fig. 7f). The relative expression of MYH9 mRNA was upregulated at 6 hpi and then reached a peak at 24 hpi (2.52-fold) following N-PRRSV infection (Fig. 7g). These results suggest that the downregulation of let-7f-5p likely upregulates MYH9 expression in PRRSV-infected PAMs.

### PRRSV replication is suppressed by overexpression of let-7f-5p

**Figure 8:**
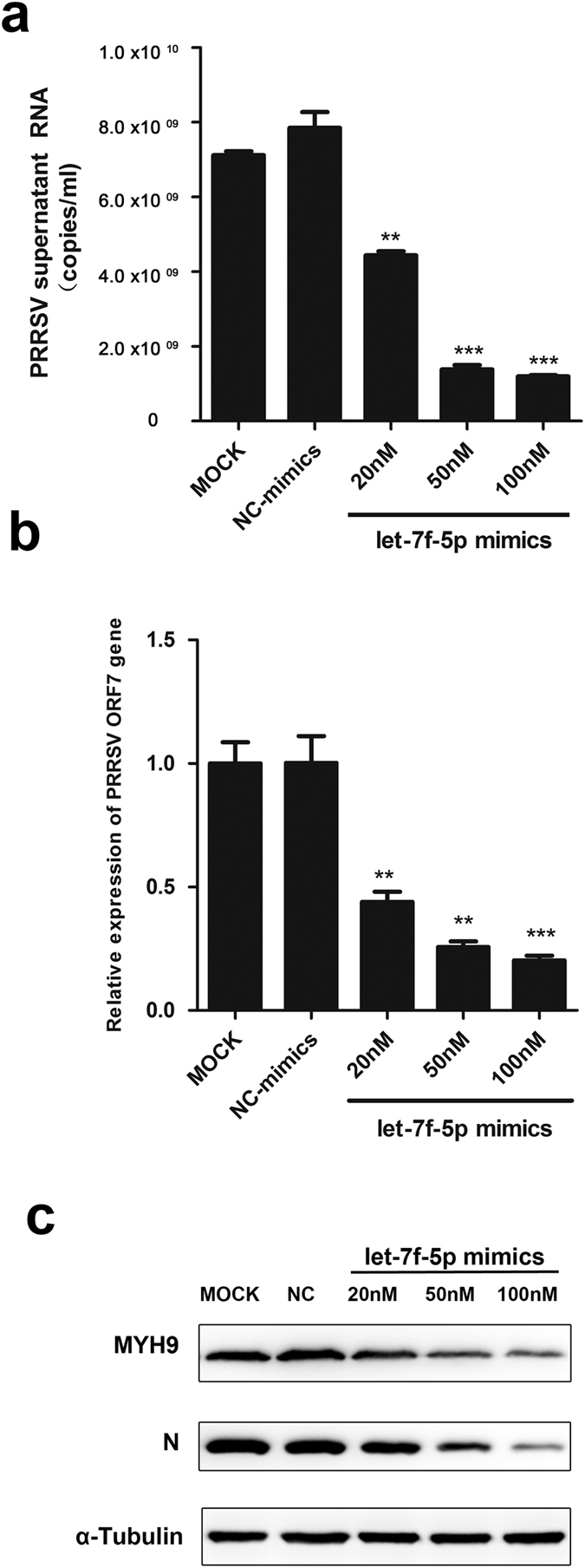
Characterization of the anti-PRRSV activity of let-7f-5p. PAMs were transfected with NC mimics or various concentrations of let-7f-5p and infected with PRRSV strain GD-HD (MOI = 0.01) for 24 hrs. PRRSV replication was evaluated by qRT-PCR to measure levels of extracellular PRRSV genome RNA in the supernatants (**a**), intracellular PRRSV ORF7 RNA (**b**), and western blot for intracellular PRRSV N protein with α-tubulin as control (**c**). Results are expressed as mean ± SD of three independent experiments. *p* values were calculated using Student’s *t* test. **P < 0.01, ***P < 0.001.

To investigate whether the activation of let-7f-5p affects PRRSV replication through targeting of the MYH9 gene, PAMs were transfected with increasing concentrations of let-7f-5p mimics (20, 50, 100 nM) or NC mimics (100 nM), followed by infection with the GD-HD strain at an MOI of 0.01. At 24 hpi, the cells were collected to determine relative ORF7 expression. PRRSV RNA copy number in cell culture supernatants were detected by qRT-PCR and cell lysates were also analyzed by western blotting. Both PRRSV growth and ORF7 mRNA levels were inhibited in a dose-dependent manner and cells transfected with a higher dose of let-7f-5p displayed a stronger antiviral effect. Notably, 100 nM let-7f-5p could markedly inhibit both MYH9 expression and viral replication, resulting in reduction in virus copy number by 83%, ORF7 mRNA by 80%, and reduction of protein expression levels (Fig. 8a–c).

### Let-7f-5p inhibits both HP-PRRSV and N-PRRSV replication

**Figure 9:**
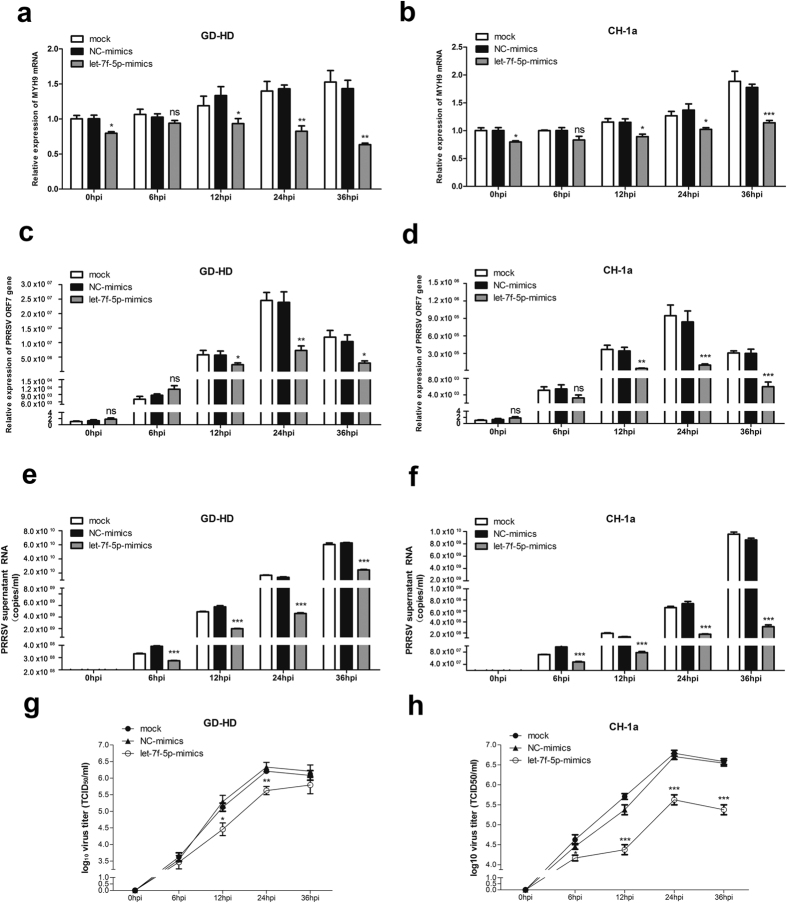
Overexpression of let-7f-5p attenuates both HP-PRRSV and N-PRRSV replication in PAMs. PAMs were transfected with 100 nM let-7f-5p or NC mimics, followed by infection with two PRRSV strains: GD-HD and CH-1a (MOI = 0.01). (**a**,**b**) Relative expression level of MYH9 mRNA was evaluated by qRT-PCR. PRRSV replication was evaluated by qRT-PCR for intracellular PRRSV ORF7 RNA (**c**,**d**) and extracellular PRRSV genome RNA in the supernatants (**e,f**), and viral titers in the supernatants were measured by TCID_50_ at the indicated time points (**g**,**h**). Results are expressed as mean ± SD of three independent experiments. *p* values were calculated using Student’s *t* test. *P < 0.05, **P < 0.01, ***P < 0.001.

To further examine viral replication inhibition caused by let-7f-5p, PAMs were transfected with 100 nM let-7f-5p mimics or NC mimics. After 24 hpi with HP-PRRSV (GD-HD) or N-PRRSV (CH-1a) (each at an MOI of 0.01), the relative expression level of MYH9 mRNA and intracellular PRRSV ORF7 mRNA, extracellular PRRSV genome RNA, and culture supernatant viral titers were detected at the indicated time points. As shown in Fig. 9a,b, significant decreases were observed in MYH9 mRNA expression after let-7f-5p transfection. Moreover, transfected PAMs contained smaller amounts of PRRSV ORF7 mRNA at 12 hpi, 24 hpi, and 36 hpi, and lower virus genome copy numbers at 6 hpi, 12 hpi, 24 hpi, and 36 hpi in both GD-HD- and CH-1a-infected PAMs (Fig. 9c–f). The inhibition observed for virus titers, as measured by TCID_50_, was significantly reduced in let-7f-5p-transfected GD-HD-infected PAMs at 12 hpi and 24 hpi; viral titers in the supernatant decreased by 0.83 and 0.71 log10 relative to control cell titers at 12 hpi and 24 hpi, respectively (Fig. 9g). A similar inhibition of CH-1a replication was observed in let-7f-5p-overexpressing PAMs, where viral titers in the supernatants decreased by 0.29, 1, 1.08, and 1.17 log10 relative to control cell titers at 6 hpi, 12 hpi, 24 hpi, and 36 hpi, respectively (Fig. 9h). Together, these results are consistent with the interpretation that let-7f-5p inhibits both HP-PRRSV and N-PRRSV replication in PAMs and the inhibitory effect induced by let-7f-5p lasts for at least 36 hpi.

## Discussion

*MYH9*, the gene for non-muscle myosin heavy chain 9, codes for a protein that exhibits multiple functions, including roles in cytokinesis, cell motility, and maintenance of cell shape^24^. A recent report demonstrates that MYH9 plays a key role in some virus infections. For example, herpes simplex virus 1 (HSV-1), Epstein-Barr virus (EBV), and PRRSV have been known to utilize MYH9 as a functional receptor that interacts with viral proteins^15^,^25^,^26^. Our previous work has shown that the MYH9 expression level apparently increased after PRRSV incubation with host cells^15^. Therefore, we hypothesized that regulatory factors control MYH9 expression.

MicroRNAs belong to a class of small non-coding RNAs which can modulate gene expression across a wide spectrum of biological systems^16^. MicroRNAs have emerged as important regulators in the complex interactions of host and virus at the posttranscriptional level^27^,^28^. Moreover, a number of cellular miRNAs have been found to regulate PRRSV infection through a variety of pathways^20^,^21^,^22^.

In the present study, we sought to determine which miRNAs could regulate MYH9 expression at the posttranscriptional level, while also regulating PRRSV replication. Using bioinformatics prediction and experimental verification, we demonstrated that let-7f-5p is a key regulator of MYH9. Results of luciferase activity assays showed that let-7f-5p regulated luciferase expression by targeting both pig and monkey MYH9 3′UTR (Fig. 1). To identify let-7f-5p targets regulated through base pairing between the miRNA “seed region” and the 3′UTR of the pig and monkey MYH9, the microRNA-mRNA relationship was validated using luciferase activity assays (Figs 2 and 3) and Ago2-IP analysis (Fig. 4). In addition, let-7f-5p and its target sites are both conserved among different host species (Figs 1c and 4a). The results show that MYH9 is a direct target for let-7f-5p and that let-7f-5p-mediated suppression of MYH9 is dependent on the MYH9 3′-UTR sequence.

Previous studies indicated that regulation of gene expression by miRNAs can be explained by two mechanisms that result in either degradation of mRNA or suppression of translation^29^,^30^. Many miRNAs regulate expression of target genes using both pathways. For example, miR-24-3p downregulates HO-1 expression through both mRNA degradation and translational repression in MARC-145 cells^20^. Our results showed that overexpression of let-7f-5p in PAMs significantly downregulated both mRNA and protein levels of MYH9 (Fig. 5). MYH9 mRNA and protein levels were augmented in cells transfected with let-7f-5p inhibitor compared to cells transfected with control inhibitor (Fig. 6). These results suggest that let-7f-5p might regulate MYH9 expression through both mRNA degradation and translational repression.

For successful establishment of infection within the host, intracellular pathogens such as viruses need to evade the host-immune response, reshape the cellular environment, and utilize host resources to synthesize viral proteins and RNA^31^,^32^. In the hide-and-seek game between virus and host, viruses have evolved highly sophisticated mechanisms for their propagation and survival^28^,^33^,^34^. Increasing evidence suggests that viral infection can influence an expression profile of host miRNAs, either through inhibition of defensive host signaling against viral infection or by viral hijacking of host defenses to favor infection^28^,^35^. The let-7 family contains some members with highly conserved sequences in species ranging from worms to humans, which share several conserved mRNA targets^17^,^36^. To understand the expression patterns of let-7 family members during PRRSV infection, we profiled host miRNAs in PAMs during HP-PRRSV and N-PRRSV infections using a deep sequencing approach. PRRSV infection of PAMs by both HP-PRRSV and N-PRRSV strains downregulated the expression of let-7f-5p and several other let-7 family members, including let-7i-5p, let-7d-5p, and let-7g-5p, resulting in greater virus infectivity. Notably, although the members share the same “seed region”, let-7 family members are each unique and encoded on different chromosomes. Furthermore, their expression levels and functions differ from one another during virus infection. The expression of let-7f-5p and MYH9 was further validated by quantitative real-time PCR. PRRSV infection markedly downregulated let-7f-5p expression and upregulated MYH9 in PAMs infected with both PRRSV strains (Fig. 7). Based on the observation that let-7f-5p is downregulated during PRRSV infection, we propose that downregulation of let-7f-5p could, in a context-specific manner, offer an advantage to PRRSV. Collectively, these results demonstrate that there is an inverse correlation between the expression of let-7f-5p and expression of MYH9.

Moreover, we observed that let-7f-5p has a significant impact on PRRSV infection and this result is consistent with previous observations showing that microRNAs are small RNAs with great power; differential regulation by let-7 family miRNAs has previously been shown to participate in regulation of the immune response to many virus infections. For example, let-7b have been reported to inhibit hepatitis C virus (HCV) infectivity by interacting with the HCV genome^37^. Let-7a suppresses Kaposi’s sarcoma-associated herpesvirus (KSHV) replication through targeting of MAP4K4 signaling pathways^38^. Let-7g inhibits hepatitis B virus (HBV) preS2 protein expression and generation of viral products^39^. Our results show that let-7f-5p can inhibit viral replication of both HP-PRRSV and N-PRRSV in PAMs (Figs 8 and 9). The potential for one miRNA to regulate numerous mRNAs and for one mRNA to be targeted by multiple miRNAs suggests that there may be other host genes regulated by let-7f-5p that limit PRRSV proliferation. Further research is needed to explore this possibility.

Here we report that MYH9 is the direct target of let-7f-5p, which regulates MYH9 expression via binding to its mRNA 3′UTR, resulting in the downregulation of MYH9 expression at both the posttranscriptional and translational level. We demonstrate that overexpression of let-7f-5p can inhibit PRRSV replication through suppression of MYH9 expression. These findings reveal an example of a host microRNA that modulates PRRSV replication and also highlights how microRNAs could be used in microRNA-mediated antiviral therapeutic strategies.

## Materials and Methods

### Cells culture and virus preparation

A PRRSV-permissive cell line, African green monkey kidney cell line (MARC-145), and human embryonic kidney cell line (HEK293FT) were cultured in Dulbecco’s modified Eagle’s medium (DMEM; Life Technologies Corporation, USA) supplemented with 10% (v/v) fetal bovine serum (FBS, GE Healthcare Life Sciences, USA) and penicillin-streptomycin (100 U/ml). PAMs were isolated from 6-week-old specific-pathogen-free (SPF) piglets and maintained in Roswell Park Memorial Institute-1640 medium (RPMI-1640, Life Technologies) supplemented with 10% (v/v) FBS and 1% antibiotic-antimycotic (Life Technologies). Animal experiments were performed according to Chinese Regulations of Laboratory Animals and the approval license number was NWAFU (Shan) 20150017/05, which was approved by the Animal Care and Use Committee of Northwest A&F University.

A highly pathogenic PRRSV (HP-PRRSV) strain GD-HD (GenBank ID: KP793736.1) and a classical type PRRSV strain CH-1a (GenBank ID: AY032626) were used in this study. The PRRSV virus stocks were propagated and titrated in MARC-145 cells and then stored at −80 °C until use.

### Cellular delivery of microRNA mimics, inhibitor, or siRNAs

Cells were cultured in 24-well plates for 12 h and transfected with mimics, inhibitor, siRNAs, or negative control siRNA synthesized by GenePharma (Shanghai, China). X-tremeGENE siRNA Transfection Reagent (Roche, Basle, Switzerland) was used, following manufacturers’ instructions. Each treatment was performed in at least triplicate. The sequences of mimics, inhibitor, and siRNAs are shown in Table 2.

### Computational prediction of miRNA-mRNA target

The potential miRNA-mRNA target interactions were predicted using miRbase^40^ (http://www.mirbase.org/), Segal Lab of Computational Biology online microRNA prediction tool (http://genie.weizmann.ac.il/pubs/mir07/mir07_prediction.html), and RNAhybrid^41^ (http://bibiserv.techfak.uni-bielefeld.de/rnahybrid/).

### Plasmids construction

To construct the psiCheck-2 (Promega, Madison, WI, USA) target luciferase reporter plasmid, the 802 bp 3′UTR of pig MYH9 and 1,083 bp 3′UTR of monkey MYH9 were amplified using PCR of cDNA of PAMs and MARC-145 cells, then cloned into the 3′ UTR of the Renilla luciferase gene using the *Xho*I and *Not*I restriction sites of the psiCheck-2 vector. Target mutations were made to the “seed region” of the let-7f-5p binding site in porcine MYH9 (pMYH9) and murine MYH9 (mMYH9), then the oligonucleotides were synthesized, annealed into double strands, and cloned into the psiCheck-2 vector. The primer sequences used for psiCheck-2 luciferase reporter plasmid construction are shown in Table 3.

### Dual-luciferase reporter assay

The dual-luciferase reporter assay was performed as previously described with the following modifications^20^. In brief, HEK293FT cells were seeded into a 48-well plate containing DMEM supplemented with 10% FBS at a density of 2 × 10^4^ cells/well. Twenty-four hours later, cells were cotransfected with gene-specific luciferase reporter plasmid constructs containing full-length MYH9 3′UTRs: psiCheck-pMYH9-WT, psiCheck2-mMYH9-WT, or mutant MYH9 3′UTR psiCheck2-pMYH9-MUT. psiCheck2-mMYH9-MUT plasmids (50 ng) and let-7f-5p mimics, let-7f-5p-MUT mimics, or NC mimics (100 nM) by using X-tremeGENE siRNA Transfection Reagent (Roche, Basle, Switzerland). Thirty-six hours post-transfection, cells were lysed and luciferase expression was measured using a Synergy HT Multi-Mode Microplate Reader (BioTek, Winooski, VT, USA) using the Dual-Luciferase Reporter Assay System (Promega, Madison, WI, USA) following manufacturer’s instructions.

### RISC immunoprecipitation assays

PAMs were mock-infected or infected with the GD-HD strain at an MOI of 0.1, harvested and lysed using cell lysis buffer (1% NP-40, 150 mM NaCl, 10 mM Tris-HCl, pH 7.8 with 1 mM EDTA) containing a proteinase inhibitor cocktail (Sigma-Aldrich, St. Louis, MO, USA), and used for RISC immunoprecipitation assays at 12 hpi. Briefly, protein G Sepharose beads (Invitrogen, Carlsbad, CA, USA) were incubated with rat anti-Ago2 monoclonal antibody (clone 2E12-1C9; Abnova, Taiwan) or with anti-Flag antibody (as an isotype control) and mixed by rotation overnight at 4 °C followed by three washes with cell lysis buffer to remove non-specifically bound material. Cell lysates from PRRSV-infected or non-infected PAMs were added to the beads and incubated for 4 h at 4 °C. RISC-associated RNAs were isolated from the beads using RNAiso Plus (TaKaRa, Dalian, China). The let-7f-5p and MYH9 mRNA in the co-precipitates were quantitated using qRT-PCR.

### Small RNA deep sequencing

Three small RNA libraries were constructed by Illumina and were sequenced using a HiSeq^TM^ 2500 platform. Briefly, total RNA was extracted from the uninfected and PRRSV-infected (GD-HD and CH-1a) cells using RNAiso Plus (TaKaRa, Dalian, China) according to the manufacturer’s instructions. RNA quality and purity were verified using the Agilent 2100 Bioanalyzer (Agilent Technologies, Santa Clara, CA, USA) and were evaluated by RNase-free agarose gel electrophoresis. The low molecular weight RNA (18–30 nt) was enriched using polyacrylamide gel electrophoresis (PAGE). Next, a pair of 5′ and 3′ RNA adapters was ligated to the small RNAs ends. After reverse transcription of the RNAs, the generated cDNA library was sequenced using the Illumina HiSeq^TM^ 2500 sequencing platform (Gene Denovo Guangzhou, China). Sequencing analysis was done using an Illumina Genome Analyzer (Illumina, CA, USA), following manufacturer’s instructions.

### Quantitative reverse transcription-PCR (qRT-PCR)

Total RNA was extracted from cells or cell culture supernatant using RNAiso Plus (TaKaRa, Dalian, China), following manufacturer’s instructions. To measure the expression of let-7f-5p, total cell RNA molecules were reverse transcribed with specific stem-loop RT primers and reverse transcriptase, then the quantitative real-time PCR was performed using a Step One Plus^®^ Real-Time PCR System (Applied Biosystems, Foster City, CA, USA) with FastStart Universal SYBR Green Master (Roche, Basle, Switzerland). The primers used for qPCR amplification are listed in Table 2 and the U6 endogenous control served as an internal standard for quantification. Use the following conditions for the qRT-PCR reactions: 95 °C for 10 min, 40 cycles of denaturation at 95 °C for 15 s, and annealing and elongation at 60 °C for 30 s. Fluorescence emission was measured at the end of each extension step. Relative expression was analyzed using the comparative threshold cycle (Ct) method.

To detect relative PRRSV *N* and *MYH9* mRNA levels, total RNA was reverse transcribed using the PrimeScript RT reagent Kit (TaKaRa, Dalian, China) with a mixture of oligo (dT) and random primers, following manufacturer’s instructions. Quantitative real-time PCR was performed as described above. The fold changes in target gene expression levels were determined using the comparative threshold cycle (Ct), with *HPRT1* as reference (housekeeping control gene). The primers used for qRT-PCR amplification are listed in Table 2.

To determine supernatant PRRSV genome RNA copy numbers, a 372 bp fragment of the PRRSV ORF7 sequence was cloned into the T-tagged site of the pMD18-T vector (TaKaRa, Dalian, China), following manufacturer’s instructions. Standard curves were generated from serial ten-fold dilutions of the plasmid constructs. PRRSV RNA genome absolute quantities were calculated by normalization to the standard curve.

### Western blot analysis

PRRSV structural protein N or cellular MYH9 protein were detected by western blotting. Briefly, cells were lysed in NP-40 lysis buffer supplemented with protease and phosphatase inhibitor. Proteins were separated on SDS-PAGE gels and then electro-transferred onto polyvinylidene fluoride (PVDF) membranes. The membranes were blocked with 5% milk in phosphate buffered saline (PBS) with Tween-20 (PBST containing 0.025% Tween-20) at 4 °C for 2 h, and then blotted with the following primary antibodies: mouse anti-PRRSV N protein monoclonal antibody (6D10), mouse antibodies against MYH9 (Sigma-Aldrich, St. Louis, MO, USA), or anti-α-tubulin antibody (Abcam, Cambridge, MA, USA) for 2 h at room temperature. After washing with PBST three times, the blots were subsequently incubated with horseradish peroxidase (HRP)-conjugated goat anti-mouse IgG (Jackson Laboratories, West Grove, PA, USA) as the secondary antibody. Finally, the proteins were visualized using enhanced chemiluminescence (ECL) reagent (Pierce, Rockford, IL, USA), following manufacturer’s instructions.

### Virus titration

To determine cell culture supernatant virus titers, MARC-145 cells were seeded into 96-well plates before virus infection. Cells grown to 80% confluence were infected with serial dilutions of each supernatant containing virus progeny in DMEM and added to each well in replicates of eight. After 2–3 days of incubation at 37 °C, cytopathic effect (CPE) was observed microscopically. The 50% tissue culture infectious dose (TCID_50_) assay end point was calculated using the Reed-Muench method.

### Statistical analysis

Statistical analysis was performed using GraphPad Prism version 5.0 (GraphPad Software, San Diego, CA, USA) and significant differences between the two groups were determined using two-tailed Student’s t-tests. *P* < 0.05 was considered statistically significant.

## Additional Information

**How to cite this article**: Li, N. *et al*. MicroRNA let-7f-5p Inhibits Porcine Reproductive and Respiratory Syndrome Virus by Targeting MYH9. *Sci. Rep.* **6**, 34332; doi: 10.1038/srep34332 (2016).

## Figures and Tables

**Table 1:** Let-7 family miRNAs detected post-PRRSV infection compared with controls.

miR name	total	Sequence reads (TPM)			Fold change	
		Control_TPM	GD-HD_TPM	CH-1a_TPM	GD-HD/Control	CH-1a/Control
ssc-let-7a-5p	224867	30750.2034	33446.7287	33861.6891	0.12126916	0.139057987
ssc-let-7f-5p	53844	9239.8717	6377.8874	6178.0687	−0.534794192	−0.580716905
ssc-let-7g-5p	28003	4747.9806	3375.6475	3262.6541	−0.492149781	−0.541267996
ssc-let-7c-5p	26983	3827.1798	3772.0587	4055.013	−0.020929553	0.08342486
ssc-let-7d-5p	17719	2592.1679	2485.4609	2497.6783	−0.06064576	−0.053571497
ssc-let-7i-5p	4153	626.357	554.7617	578.5385	−0.175116984	−0.114572204
ssc-let-7e-5p	269	38.6494	40.1226	36.7049	0.05396908	−0.074473346

**Table 2:** The sequences of mimics, inhibitor, and siRNAs.

Name	Sequence
let-7f-5p mimics	5′-UGAGGUAGUAGAUUGUAUAGUU-3′
let-7f-5p-Mut mimics	5′-ACUCCAAGUAGAUUGUAUAGUU-3′
NC mimics	5′-AGCUGAUUUCGUCUUGGUA-3′
let-7f-5p-inhibitor	5′-AACUAUACAAUCUACUACCUCA-3′
NC inhibitor	5′-UCACCGGGUGUAAAUCAGCUUGAC-3′
MYH9 siRNA	Sense: 5′-dTdTGACAGAAUAGCUGAGUUCA-3′
	Antisense: 3′-CUGUCUUAUCGACUCAAGUdTdT-5′
NC-siRNA	Sense: 5′-dTdTUGCACUGUGCAAGCCUCUU-3′
	Antisense: 3′-ACGUGACACGUUCGGAGAAdTdT-5′

**Table 3:** Primers used for qRT-PCR and psiCheck2 vector construction.

Primer name	Sequence
let-7f-RT-Primer	5′-CTCAACTGGTGTCGTGGAGTCGGCAATTCAGTTGAGAACTATA-3′
let-7f-Stem-Loop-F	5′-ACACTCCAGCTGGGTGAGGTAGTAGATTGTA-3′
let-7f-Stem-Loop-R	5′-CTCAACTGGTGTCGTGGAGTCGGCAATTCAG-3′
U6-RT-Primer	5′-CTCAACTGGTGTCGTGGAGTCGGCAATTCAGTTGAGAAAAATATGG-3′
U6-Stem-Loop-F	5′-CTCGCTTCGGCAGCACA-3′
U6-Stem-Loop-R	5′-AACGCTTCACGAATTTGCGT-3′
pMYH9-qF	5′-AAGGCACCGTCAAGTCCAA-3′
pMYH9-qR	5′-TTCCTCCGCTCATCATCCA-3′
PRRSV-ORF7-gene-F	5′-AGATCATCGCCCAACAAAAC-3′
PRRSV-ORF7-gene-R	5′-GACACAATTGCCGCTCACTA-3′
HPRT1-F	5′-TGGAAAGAATGTCTTGATTGTTGAAG-3′
HPRT1-R	5′-ATCTTTGGATTATGCTGCTTGACC-3′
psiCheck2-mMYH9-3UTR-F	5′-TTACTCGAGGCCTCTTCTCCTGCAGCCT-3′
psiCheck2-mMYH9-3UTR-R	5′-TTGCGGCCGCTTTTTGGTAGCACATGGTTCTCTT-3′
psiCheck2-pMYH9-3UTR-F	5′-TTACTCGAGCAGCCTGCTCTTCTCCTGCT-3′
psiCheck2-pMYH9-3UTR-R	5′-TTGCGGCCGCTGTTGGGAGAAGCCACGGCT-3′
psiCheck2-mMYH9-3UTR-Mut-F	5′-TCGAGTGCTGTTGGCAATCACACGTGGTGTGGAGTCACACCTCTGCCCCTTGGGCCGC-3′
psiCheck2-mMYH9-3UTR-Mut-R	5′-GGCCGCGGCCCAAGGGGCAGAGGTGTGACTCCACACCACGTGTGATTGCCAACAGCAC-3′
psiCheck2-pMYH9-3UTR-Mut-F	5′-TCGAGTGCTGTTGGCAATCACACGCGGTGTGGAGTCACCCTCTGCCCCCCCCAGCCGC-3′
psiCheck2-pMYH9-3UTR-Mut-R	5′-GGCCGCGGCTGGGGGGGGCAGAGGGTGACTCCACACCGCGTGTGATTGCCAACAGCAC-3′
